# Directed Self-Assembly of Polystyrene Nanospheres by Direct Laser-Writing Lithography

**DOI:** 10.3390/nano10020280

**Published:** 2020-02-07

**Authors:** Eleonora Cara, Federico Ferrarese Lupi, Matteo Fretto, Natascia De Leo, Mauro Tortello, Renato Gonnelli, Katia Sparnacci, Luca Boarino

**Affiliations:** 1 Advanced Materials Metrology and Life Sciences Division, Istituto Nazionale di Ricerca Metrologica, Strada delle Cacce 91, 10135 Torino, Italy; e.cara@inrim.it (E.C.); m.fretto@inrim.it (M.F.); n.deleo@inrim.it (N.D.L.); l.boarino@inrim.it (L.B.); 2 Dipartimento di Scienza Applicata e Tecnologia (DISAT), Politecnico di Torino, Corso Duca degli Abruzzi 24, 10129 Torino, Italy; mauro.tortello@polito.it (M.T.); renato.gonnelli@polito.it (R.G.); 3 Dipartimento di Scienze e Innovazione Tecnologica (DIST), Universitá del Piemonte Orientale “A. Avogadro”, INSTM, Viale T. Michel 11, 15121 Alessandria, Italy; katia.sparnacci@uniupo.it

**Keywords:** directed self-assembly, nanospheres lithography, colloidal nanospheres, direct laser-writing

## Abstract

In this work, we performed a systematic study on the effect of the geometry of pre-patterned templates and spin-coating conditions on the self-assembling process of colloidal nanospheres. To achieve this goal, large-scale templates, with different size and shape, were generated by direct laser-writer lithography over square millimetre areas. When deposited over patterned templates, the ordering dynamics of the self-assembled nanospheres exhibits an inverse trend with respect to that observed for the maximisation of the correlation length ξ on a flat surface. Furthermore, the self-assembly process was found to be strongly dependent on the height (H) of the template sidewalls. In particular, we observed that, when H is 0.6 times the nanospheres diameter and spinning speed 2500 rpm, the formation of a confined and well ordered monolayer is promoted. To unveil the defects generation inside the templates, a systematic assessment of the directed self-assembly quality was performed by a novel method based on Delaunay triangulation. As a result of this study, we found that, in the best deposition conditions, the self-assembly process leads to well-ordered monolayer that extended for tens of micrometres within the linear templates, where 96.2% of them is aligned with the template sidewalls.

## 1. Introduction

Nanospheres lithography (NSL) is a manufacturing technique based on the self-assembly (SA) process of colloidal spheres [1]. Monodisperse suspensions of polystyrene (PS) nanospheres (NSs) deposited on a substrate form colloidal crystals consisting in single or multiple layers, exhibiting hexagonal close-packed (HCP) symmetry. In the last decades, NSL gained increasing attention in nanotechnology due to the possibility to realise several periodic patterns over large area and at reasonable cost, including photonic structures [2] or devices for nanoelectronics [3] and plasmonics [4]. However, the SA process exhibits intrinsic variability, resulting in the generation of lattice defects and the formation of multiple domains. These irregularities hinder advanced applications in which precise spatial positioning of the nanostructures is required. In this context, the design of an experimental procedure with stable output is demanded for the fabrication of well-ordered single domains with controlled size and regular shape.

An interesting solution to overcome this limitation is represented by the use of substrate modifications to aid the formation of single-layered crystals of NSs. This approach, also called directed self-assembly (DSA), has been successfully proposed for other self-assembling systems, such as Block Copolymers (BCPs), receiving great consideration so far due to its wide applicability in key technological sectors such as microelectronics [5,6,7]. The substrate can be modified either by a chemical [8,9] or topographic templates generated prior the SA process [10]. In the latter case, the bottom-up SA process is directed by the presence of confining structures such as linear or circular gratings, defined by conventional top-down lithographic approaches [11]. The geometrical dimensions of the topographic templates can be tailored to be commensurate to the characteristic dimensions of the SA material (e.g., diameter of the NSs or center-to-center distance for BCPs).

The development of DSA processes applied to NSL has been mainly dedicated to the confinement of few NSs [12,13,14] or to achieve size separation of polydispersed NSs [15]. The present work aims to extend the DSA process over large area, to allow the formation of single-grain domains highly oriented inside pre-patterned templates throughout a several square millimetre area. To meet this objective, direct laser writing (DLW) lithography and reactive ion etching (RIE) were combined to fabricate micrometric templates with different shapes and sizes. The deposition of the NSs in the templates was performed by spin coating, and the dynamic parameters were varied starting from the insights of our previous work [16]. In particular, we investigate the formation of the NSs monolayer through the analysis of the confinement and the ordering processes. The former was carried out by means of atomic force microscopy (AFM) and scanning electron microscopy (SEM), whereas the NSs ordering was evaluated through an image-processing method measuring the domains orientation. These analyses contribute to increase the repeatability of NSL and expand its applicability through DSA to address the necessities of the development of novel devices for photonics [17], chemical sensing [18,19], data storage [20], and optoelectronics [21].

## 2. Materials and Methods

### 2.1. Direct Laser-Writing Patterning

**Figure 1:**
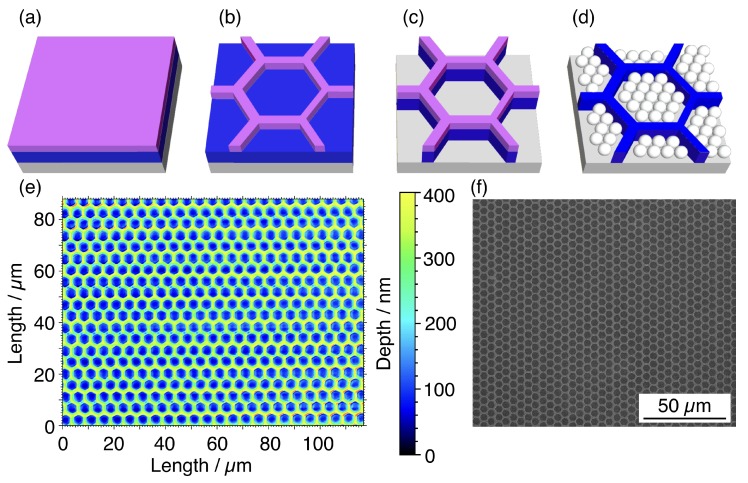
(**a**) The silicon (grey) substrate with a $SiO_{2}$ layer (blue) is covered with a layer of photosensitive resist (purple). (**b**) The hexagonal pattern is designed on the resist by direct laser writing (DLW) lithography and developed to expose the underlying $SiO_{2}$ layer, (**c**) which is then etched by reactive ion etching process (RIE). (**d**) The PS NSs are deposited inside the resulting template. (**e**) Optical profilometry image and (**f**) SEM micrograph at low magnification showing the hexagonal templates over large area after the etching.

The DLW lithography (Heidelberg μPG101 laser writer, Heidelberg, Germany) was performed on polished silicon wafers (MEMC Electronic Materials, Novara, Italy) covered by a thermal oxide layer with thickness ranging between 50 nm and 200 nm. An optical resist (AZ 1505 Merck Performance Materials GmbH, Darmstadt, Germany) was deposited over the $SiO_{2}$ substrate (Figure 1a) and exposed with a laser beam (λ=375nm, diameter of 800 nm and intensity of 10 mW). The resist was afterward developed for 40 s in a 1:1 solution of the developer (AZ Developer Merck Performance Materials GmbH) and $H_{2}O$. The resulting pattern left the $SiO_{2}$ layer exposed, as shown in Figure 1b. The templates sizes were designed to confine an integer number of NSs, including hexagonal templates with a diagonal length of 4.75 μm and linear ones with a width of 3 μm and length of 200 μm, while Figure 1 only reports the hexagonal configuration as an example.

### 2.2. Template Fabrication

The DLW pattern was transferred to the oxide layer by reactive ion etching process (RIE) (Figure 1c). The chemically reactive plasma was obtained by mixing $CHF_{3}$ and Ar with a flow ratio of 54 sccm to 29 sccm. The plasma was generated with a residual pressure of 180 Pa and an applied RF power of 300 W, with a typical reflected power of 25 W. Under these operating conditions, the etching rate on $SiO_{2}$ was 10 nm min^−1^ and the time was selected to reach different depths. After the etching step, the excess resist was removed with acetone and the final patterned substrate was characterised by a non-contact 3D surface profiler (Sensofar S Neox, Barcelona, Spain) (Figure 1e) and a field emission gun (FEG) SEM (FEI Inspect-FTM, Hillsboro, OR, USA) (Figure 1f).

### 2.3. Nanospheres Deposition

Despite the other NSs deposition methods that have been proposed so far, such as doctor blading [22] or Langmuir–Blodgett coating [23], in this work, we used the spin coating technique. Such choice was motivated by the aim to develop proper protocols to promote the applicability of DSA processing in industrial nanomanufacturing already relying on this method. The patterned substrates were cleaned in an ultrasonic bath of acetone and isopropyl alcohol. The surface was treated by $O_{2}$ plasma for 6 minutes at 40 W with a residual pressure of 3 Pa to make it hydrophilic. The PS NSs were synthesised using the emulsion polymerisation of styrene using sodium dodecyl sulfate as surfactant and potassium persulfate as the initiator [19]. The NSs had diameter equal to (250±4) nm and presented negative charges at the surface, due to the decomposition of the initiator, thus stabilising the aqueous suspension against aggregation. We drop coated all the samples with 60 μL of the suspension and spread it by spin coating (WS-400B-6NPP/LITE Laurell Technologies, North Wales, PA, USA) in two steps. In the first step, we set the speed and acceleration to 500 rpm and 410 rpm/s, respectively, and the duration to 10 s. For the second step, we modified the spinning speed to test the confinement process while keeping the duration at 30 s. An illustration of the result is shown in Figure 1d.

### 2.4. SEM Characterisation and Image Processing

The characterisation of NSs self-assembly inside the templates was performed by a systematic analysis of the SEM micrographs. We set conditions for the SEM imaging with V=10 kV, planar configuration at the optimum working distance of 10 mm and magnification of 10,000. For a quantitative analysis of the DSA process, we processed the images by means of a MATLAB routine which operates by recognising the NSs inside the templates and by mapping the lattice according to Delaunay triangulation. Then, it identifies deviations from the ideal HCP lattice by counting the number of nearest neighbours to each particle. The orientation of all the unit HCP cells is extrapolated with an angular resolution of 1° in the range of possible orientations of the crystals between −30° and 30°. A complete description of the operating principle of the software is reported in reference 16.

### 2.5. Atomic Force Microscopy Characterisation

The surface topography on the NSs soft material was investigated by means of atomic force microscopy (Bruker Corp. INNOVA microscope) by using etched Si probes (Bruker RTESPA-300, Billerica, MA, USA) with nominal spring constant of 40 N m^−1^ and tip radius of 8 nm. The measurements were performed in tapping mode with a resonance frequency of 230 kHz and scanning rate of 0.5 Hz. The analysis of the AFM micrographs was carried out by the freeware Gwyddion. The plane inclination was corrected by fitting a plane through three points on the optically flat $SiO_{2}$ mesas and by setting the scale zero position at the same level.

## 3. Results and Discussion

### 3.1. Nanospheres Ordering

**Figure 2:**
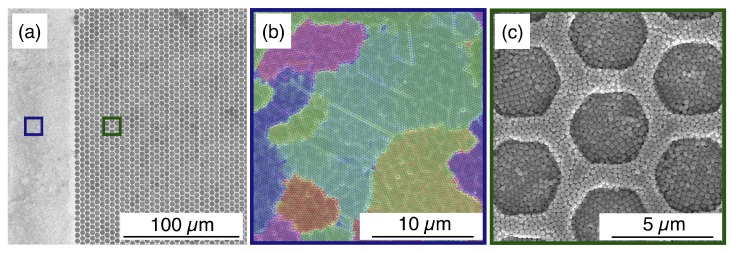
(**a**) Low-magnification SEM micrograph preliminarily comparing the SA of NSs on the flat and patterned area of the substrate. The blue and green frames correspond to the high-magnification SEM image (**b**) of the self-assembled monolayer, where the domains are highlighted in different colours showing a random change in the orientation from one to the other, and (**c**) inside the templates, where multiple layers are formed.

The deposition of NSs over the patterned substrates was performed by spin-coating process. We set the spinning speed and acceleration to 1250 rpm and 410 rpm/s, in agreement to our previous experiments focused on the maximisation of the degree of order, expressed in terms of correlation length ξ [16], on flat unpatterned substrates. Figure 2a shows a low-magnification SEM micrograph of both flat and patterned areas on the substrate. On the flat portion of the sample, the formation of large grains is preserved, as highlighted in Figure 2b by the overlapped colour map. Each coloured region corresponds to a grain or domain in which the orientation of the HCP lattice is uniform, whereas it varies randomly in the neighbouring domains separated by the grain boundaries. However, the same spinning conditions were found inadequate for the SA inside the templates, leading to the accumulation of NSs in multiple layers reported in Figure 2c.

**Figure 3:**
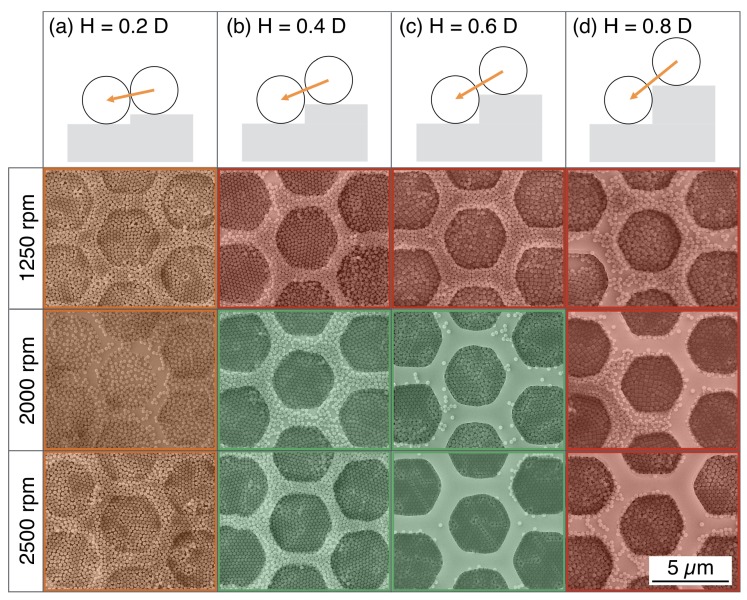
Tabular comparison of the SEM images of NSs assemblies inside hexagonal templates. The height of the confining wall varies along the columns, column (**a**) H = 0.2·D, column (**b**) H = 0.4·D, column (**c**) H = 0.6·D and column (**d**) H = 0.8·D. The spin-coating speed is varied in the rows from 1250 rpm to 2500 rpm. The SEM images highlighted in red and orange refer to unsuitable confinement conditions, whereas those coloured in green display suitable confinement.

This preliminary result highlights the differences of the SA induced on a flat substrate and inside the templates. The SA process has been described in literature as the interaction of capillary forces between two adjacent NSs, responsible for the hexagonal packing. In the presence of a geometrical constraint, such capillary forces also act across the edges of the templates, which introduce a perturbation of the conventional SA process [13,24,25]. To quantify the effect of the perturbation on the long-range ordering and to optimise the confinement of the NSs, we realised a new set of samples by varying both the height of the sidewalls and the spinning conditions. In particular, the spinning speed were set to 1250 rpm, 2000 rpm and 2500 rpm, whereas the selected heights H were 50 nm (H = 0.2·D), 100 nm (H = 0.4·D), 150 nm (H = 0.6·D) and 200 nm (H = 0.8·D). The maximum value of H (i.e., 200 nm) was chosen below the NSs diameter since excessive height would result in a physical barrier promoting the stratification in multiple layers. Figure 3 reports a tabular comparison of the SEM micrographs of the colloidal crystal, where the sidewalls height and spinning speed are varied along the columns and rows, respectively. The structures were patterned with hexagonal shape for its similarity to the characteristic packing symmetry of the NSs.

In the case of templates with H = 0.2·D (i.e., depth of 50 nm) shown in Figure 3a, the NSs self-assemble into monolayers irrespective on spinning speed. However, under these particular conditions, the orientation of the domains is not influenced by the presence of the template, as testified by the formation of grains with same orientation across the edges. For this reason, these conditions are not proper for NSs confinement and the corresponding images are coloured in orange.

On the contrary, in templates with H = 0.4·D and H = 0.6·D in Figure 3b,c, the arrangement of the NSs presents a marked dependence on the spinning parameters. For depositions performed at 1250 rpm, we observed the formation of multiple layers inside the templates (red images in Figure 3), preventing the lithographic use of the confined NSs. Such an issue can be solved by increasing the spinning speed to 2000 rpm and 2500 rpm. In this case, the NSs arrange in a single layer confined inside the templates and, despite the presence of residual NSs on the mesas in between adjacent templates, no domains are continuously ordered across the edges. In these conditions, the formation of the monolayer is facilitated and visibly influenced by the presence of the templates, the corresponding micrographs are coloured in green in Figure 3. Finally, when deposited in templates with H = 0.8·D (i.e., depth of 200 nm), the NSs accumulate in multiple layers independently of the spin-coating speed so that these conditions are not suitable for lithographic purposes (SEM images coloured in red in Figure 3d). In light of this result, the structures with H/D ratios of 0.2 and 0.8 seems to be either too shallow or too deep to produce proper confinement of the NSs. On the other hand, the structures with H equal to 0.4 or 0.6 times the NSs diameter promote the formation of confined and ordered monolayers at 2000 rpm and 2500 rpm.

**Figure 4:**
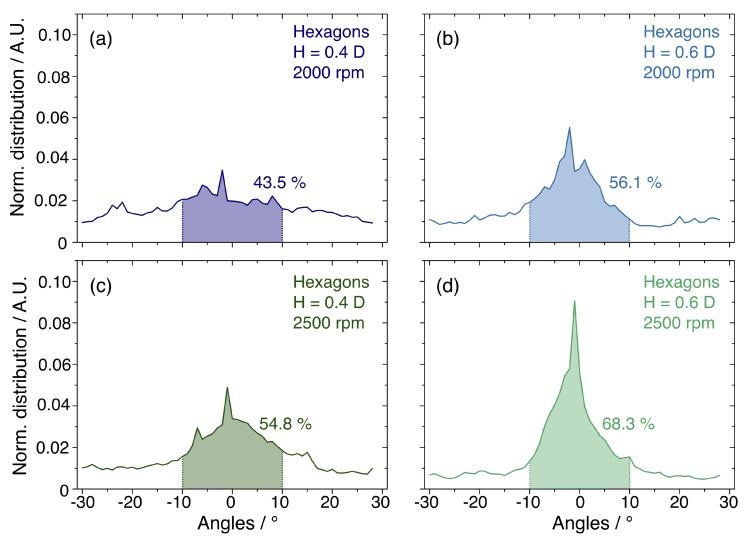
Line graphs reporting the normalised distribution of the domains orientation in the NSs monolayer confined in hexagonal graphoepitaxy structures with (**a**) H = 0.4·D at 2000 rpm, (**b**) H = 0.6·D at 2000 rpm, (**c**) H = 0.4·D at 2500 rpm and (**d**) H = 0.6·D at 2500 rpm. The orientation is evaluated in the angular range between −30° and 30°. The percentage of domains aligned to the templates edges is calculated through the integral of the curve in the range from −10° to 10° and the area and percent value are reported for each curve.

So far, the selection of the optimal self-assembly parameters has been based on a qualitative analysis of the SEM images. To establish the efficiency of the DSA of NSs inside the hexagonal templates in a more rigorous way, the ordering process should be assessed quantitatively. To this goal, the SEM micrographs were processed with a user-defined image-processing routine based on Delaunay triangulation, measuring the orientation of HCP domains. The software recognises the domains and classify them according to their rotations in the angular range between −30° and 30°. The analysis was conducted on the hexagonal templates with H/D ratios of 0.4 and 0.6 highlighted in green in Figure 3. The results are collected in Figure 4, and report the normalised distributions of the orientation of the confined monolayer in different geometrical and dynamic conditions. Such angular distributions are centred on 0° indicating an alignment to the templates edges, while slight deviations in the orientation broaden the distributions. These can be accounted for by calculating the integral of the curve which gives the percent occurrence of the domains in a given orientation range. In the hexagonal templates with H/D ratio of 0.4 and 0.6, spinning speed of 2000 rpm lead to 43.5% and 56.1% of domains with orientation comprised between −10° and 10°, as shown in Figure 4a,b, respectively. In the graphs in Figure 4c,d, the percentage of aligned domains increases to 54.8% and 68.3% when the spin coating speed is set to 2500 rpm for H = 0.4·D and H = 0.6·D, respectively. This quantitative result clearly highlights that templates with H = 0.6·D induce a better ordering of the NSs when deposited at high spinning speed.

**Figure 5:**
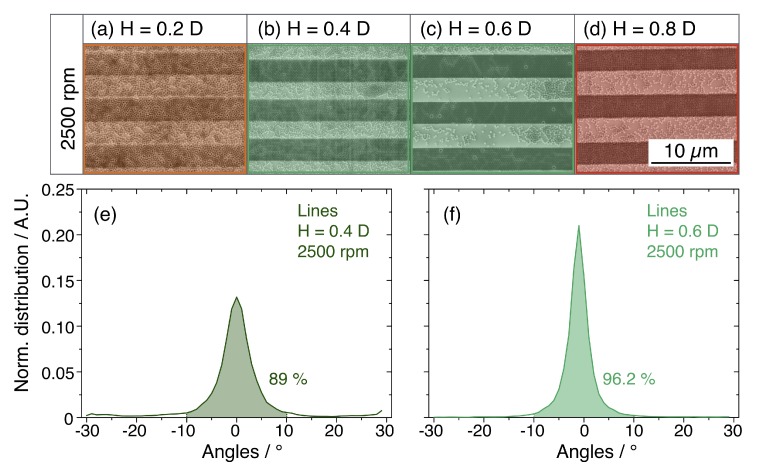
(**a**–**d**) SEM images of the NSs assemblies inside linear templates. The height of the constraining walls is varied from 0.2 to 0.8 times the diameter of the NSs across the images. (**e**,**f**) Line graphs reporting the normalised distribution of the domains orientation. The highlighted area corresponds to the percentage of domains aligned to the templates sidewalls within ±10°.

The confinement process was tested also inside linear templates, chosen for its simple realisation by DLW lithography, using the same optimal spinning conditions. The SEM micrographs reported in Figure 5a–d show the outcome of the SA process in the linear templates. Similarly to what was observed for the hexagonal templates, the micrographs coloured in orange (Figure 5a) and red (Figure 5d) correspond to unsuitable conditions for the DSA. Conversely, the templates with H/D ratio of 0.4 and 0.6 promote the formation of a confined self-assembled monolayer, as shown in Figure 5b,c. Also, in this case, the SEM micrographs were processed by Delaunay triangulation to evaluate the ordering process in terms of the domains orientation. The results of this study, reported in the graphs in Figure 5e,f, outline an angular distributions centred on 0° with narrow peaks including 89% of domains in the range from −10° and 10° for H = 0.4·D. This percentage rise up to 96.2% inside structures with H = 0.6·D.

According to this result, the linear templates induce a finer orientation constraint than the hexagonal ones, as they presented a regular shape and uniform width along their length as visible in Figure 5. On the other hand, the hexagonal structures presented some rounded features that may constitute a cause for the lower quality of the ordering process. Moreover, the dimension of the templates may differ from the pattern design causing incommensurability and the generation of defects in the colloidal lattice.

### 3.2. Nanospheres Confinement

Although the optimisation of the geometry and process parameters have led to a good result in terms of NSs ordering within the templates, the confinement process can be further investigated by considering the defectivity at the edge of the templates and the presence of residual nanospheres on the mesas, observed in Figure 3 and Figure 5. We performed an AFM analysis of the confinement process focusing our attention on the height profile of the confined nanospheres in the two studied morphologies (i.e., hexagonal and linear) with H = 0.6·D.

**Figure 6:**
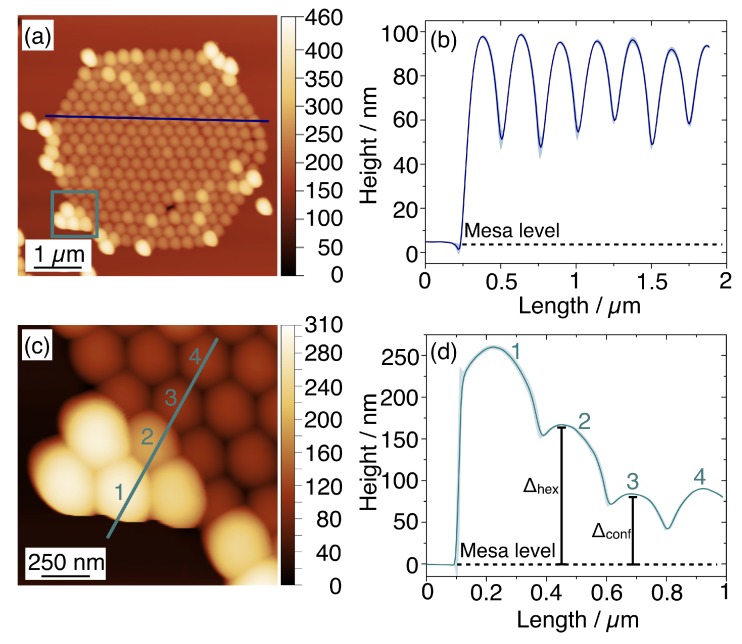
(**a**) AFM micrograph acquired on a hexagonal confining structure filled with a monolayer of NSs. The blue line marks the height profile in the line graph (**b**), where the shadowed region indicates the uncertainty of three repeated measurements. (**c**) The second AFM topographic image is acquired in the framed area in (**a**) and the line profile and numbered nanospheres are indicated. (**d**) The corresponding height profile reports nanosphere #2 is found at a higher level with respect from the mesa, $\Delta_{\mathrm{hex}}=\left(167\pm 1\right)$ nm, with respect to NSs #3 and #4.

Figure 6a reports an AFM map acquired on the hexagonal template. The height profile in Figure 6b indicates that, when good confinement is achieved, the NSs are perfectly aligned inside the hexagonal structure and exceed the mesa by $\Delta_{\mathrm{conf}}=\left(96\pm 4\right)$nm. This value is quite similar to the one expected for H = 0.6·D as the difference between the sidewalls height and the sphere diameter. The AFM maps acquired in proximity of a defect (Figure 6c) and the corresponding height profile (Figure 6d) show an irregular arrangement of NSs. The nanosphere #2, closest to the confining wall, is found at the level $\Delta_{\mathrm{hex}}=\left(167\pm 1\right)$nm above the mesa structure, whereas the NSs #3 and #4 are correctly confined at the level $\Delta_{\mathrm{conf}}=\left(90\pm 5\right)$ nm.

**Figure 7:**
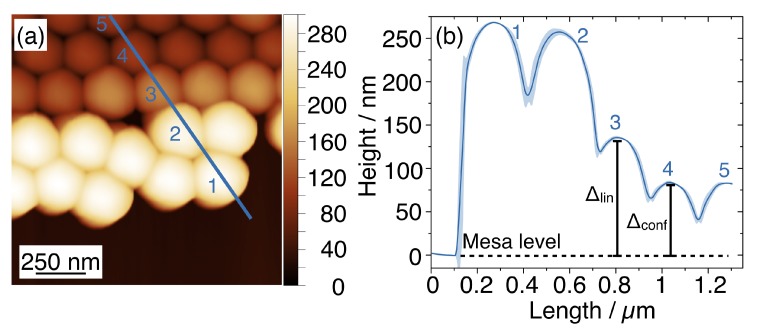
(**a**) AFM micrograph acquired on the NSs monolayer inside a linear template. The coloured line indicates the height profile evaluated on the topographic image. (**b**) The corresponding line graph presents the height variation as a function of lateral displacement. The value of the distance between NSs #3 and the top of the mesa is reported as $\Delta_{\mathrm{lin}}=\left(136\pm 2\right)$ nm.

Figure 7a,b reports the AFM micrograph acquired on a linear template and the corresponding height profile, respectively. When the NSs are well confined inside the template (e.g., the NSs labelled as #4 and #5), they lay at the same level for which $\Delta_{\mathrm{conf}}=\left(86\pm 2\right)$nm. By approaching the side walls, the height of the nanospheres increases and NS #3 is separated from the top of the mesa by $\Delta_{\mathrm{lin}}=\left(136\pm 2\right)$nm.

From this analysis, we observed the top of well-confined nanospheres to be at the level $\Delta_{\mathrm{conf}}$ from the mesa, approximately equal to the difference between the diameter D and the sidewalls height H. When the separation exceeded this quantity, such as for $\Delta_{\mathrm{hex}}$ and $\Delta_{\mathrm{lin}}$ larger than $\Delta_{\mathrm{conf}}$, we observed the onset of a defect and the accumulation of unconfined NSs on the mesas. Given that $\Delta_{\mathrm{lin}}$ was lower than the corresponding $\Delta_{\mathrm{hex}}$, the linear templates offered a better confinement of the nanospheres with respect to the hexagonal structures. In both templates, the observed distortions from the HCP symmetry can be due to several reasons, including local defectivity in the lithographic template, incommensurability of the graphoepitaxy structures or polydispersity of the nanospheres. These defects can be largely reduced by improving the combination of DLW lithography and RIE to obtain high regularity of the templates and fidelity to the pattern design. A possible strategy to limit the accumulation of excess NSs could be to graft hydrophobic polymer chains on the surface of the mesa.

**Figure 8:**
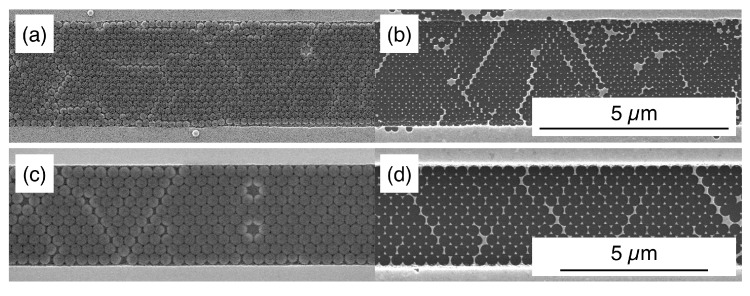
NSs with different sizes—(**a**) 200 nm and (**c**) 400 nm—are confined in a monolayer inside linear templates where the ratio H/D is kept constant at 0.6. This lithographic mask can be used for the realisation of arrays of gold nanotriangles (**b**,**d**) with tunable dimensions to match the excitation wavelengths for the sensing of different SERS-active analyte species.

Despite some local defectivity, the use of DLW lithography and RIE makes it simple to tailor the templates with H/D ratio fixed at 0.6 to confine NSs with different dimensions, as shown in Figure 8a,c for NSs with a diameter of 200 nm and 400 nm, respectively.

One common application of NSL consists in the realisation of triangular metallic nanoparticles as substrates for surface-enhanced Raman spectroscopy (SERS) applications, for the possibility to tune their geometrical features to match different excitation wavelengths [26]. DSA-NSL constitute a versatile solution to improve the uniformity and reproducibility in the fabrication of such substrates to benefit their spectroscopic responses, as it can be employed in the production of these and other metallic arrays with regular orientation and a high degree of order, as shown for example in Figure 8b,d.

## 4. Conclusions

In this work, we investigated the confinement and ordering process of the self-assembling NSs by changing the deposition parameters and the height of the confining walls in templates with two different shapes. The most appropriate conditions for the DSA-NSL where highlighted by a systematic SEM analysis correlated by the evaluation of the HCP orientation by image processing and atomic force microscopy measurements. High spinning speed of 2500 rpm were found to be necessary to let the NSs overcome the physical barriers of the templates. Sidewalls height H was found to provide proper confinement conditions at 0.6 times the NSs diameter.

DSA-NSL inside linear templates, with the previously stated geometrical and dynamic conditions, resulted in a confined monolayer aligned to the template for 96.2%. The knowledge on the DSA process and the control over the geometry through DLW lithography and RIE, allow to direct the SA of colloidal NSs to obtain single-grain crystals with uniform orientation and regular shape over large area. The optimised fabrication protocol could extend the versatility of DSA-NSL for applications requiring different geometries. The linear structures, for example, can be employed to confine the nanostructures in microfluidic channels for multiplexed analysis [27]. Moreover, hexagonal and circular structures with micrometric sizes can serve in site-specific incubation for different analytes in sensing applications, where the templates are easily recognised by optical microscopy to find the area of analysis.
